# Loneliness and Social Isolation Among Older People in Northern Ireland

**DOI:** 10.1093/geroni/igaa057.2207

**Published:** 2020-12-16

**Authors:** Charlotte Neville, Paula Devine

**Affiliations:** 1 Centre for Public Health, Queen’s University Belfast, Belfast, Northern Ireland, United Kingdom; 2 Queen’s University Belfast, Belfast, Northern Ireland, United Kingdom

## Abstract

Loneliness and social isolation are increasingly recognised as being public health concernsparticularly in older people. Social isolation can be defined as the lack of social connections, whilst loneliness is a more subjective concept and relates to negative feelings about a lack of connections. This research explores the patterns of loneliness and social isolation of over 5,000 people aged 50 years or over living in Northern Ireland who participated in the first wave of the NICOLA study (Northern Ireland Cohort for the Longitudinal Study of Ageing). Data were obtained by computer-assisted personal interviews and self-completion questionnaires. We focused on loneliness and social interaction, in relation to key demographic and socio-economic variables including age, gender and marital status. Key findings were that loneliness patterns varied according to gender, age, income, health and living circumstances. Future waves of NICOLA will help to longitudinally explore the effects of transition on loneliness and social isolation.

